# Unveiling the Heterogeneity and Multifunctions of Biological Membranes: A Unifying Perspective in Membrane Biophysics from Molecular Coupling to Cellular Function

**DOI:** 10.3390/membranes16030079

**Published:** 2026-02-24

**Authors:** Hao Wu, Zhong-Can Ou-Yang

**Affiliations:** 1 Zhejiang Key Laboratory of Soft Matter Biomedical Materials, Wenzhou Institute, University of Chinese Academy of Sciences, Wenzhou 325000, China; 2 Institute of Theoretical Physics, Chinese Academy of Sciences, Beijing 100190, China

Biological membranes are the quintessential functional interface of life, where lipid bilayer mechanics, compositional heterogeneity, and dynamic protein interactions converge to govern cellular physiology. For decades, understanding this complex subject has been a central challenge in research. This Special Issue, “*Design Principles and Biomedical Applications of Multifunctional Biological Membranes*”, presents a cohesive leap forward. The six articles within collectively address a major challenge: moving beyond the description of idealized, homogeneous membranes towards a predictive, quantitative understanding of real, heterogeneous, and active systems. This Editorial frames these contributions within the historical quest to understand membrane heterogeneity—from the seminal “lipid raft” hypothesis to modern continuum- and particle-based models. We highlight how the new theoretical framework on multicomponent membranes provides a critical, unified foundation by rigorously integrating curvature–composition coupling with essential geometric constraints, providing a solution to a longstanding limitation in the field. Subsequent articles explore the active, non-equilibrium mechanisms that exploit this heterogeneity for cellular function; the interaction of membranes with their physical environment; and the ultimate link between membrane-driven shape and cellular state. Together, they chart a course from molecular-scale design principles to tissue-level implications, marking a significant maturation of membrane biophysics into a quantitative, predictive science with profound biomedical relevance.

## 1. The Persistent Challenge of Membrane Heterogeneity

The defining feature of a living cell is its boundary—a lipid bilayer that separates the interior from the exterior world. While the self-assembly of phospholipids into vesicles provided an early, elegant model, the true biological membrane is anything but a uniform sea of lipids. More than two decades ago, the pioneering concept of “functional rafts” floating in the membrane plane fundamentally reshaped our view, introducing heterogeneity as a core design principle [1]. These dynamic, submicroscopic assemblies, enriched in sphingolipids, cholesterol, and specific proteins, are now understood to be crucial for protein sorting, signal transduction, and membrane trafficking, with their existence implying a membrane that is laterally organized, with domains of distinct composition, thickness, and mechanical properties—such as increased bending rigidity and area incompressibility—coexisting within a fluid bilayer [2,3].

This realization of inherent heterogeneity has driven the field along two complementary paths: the biochemical and biophysical dissection of domain formation [4,5], and the development of physical models capable of describing their consequences. Giant unilamellar vesicles (GUVs) have become a vital experimental workhorse, mimicking cellular membranes and revealing phenomena like budding and fission driven by lipid immiscibility or protein–lipid interactions [6]. Early experiments using fluorescence microscopy and Förster resonance energy transfer (FRET) provided direct evidence for lipid domains in model membranes [7,8,9], while later work using atomic force microscopy (AFM) and neutron scattering resolved nanoscale heterogeneity in synthetic and native membranes [10,11,12]. Concurrently, theoretical efforts have sought to extend the foundational Helfrich curvature elasticity model [13] beyond its single-component origins.

The quest for a comprehensive continuum model has been arduous. Early shape models, like the Area Difference Elasticity (ADE) model, successfully described red blood cell morphologies but treated the membrane as a homogeneous sheet [14]. The spontaneous curvature model [13] and bilayer-couple hypothesis [15] further connected membrane asymmetry to shape changes. Models incorporating membrane–skeleton coupling added biological realism but remained limited in describing composition dynamics [16]. Seminal theoretical predictions and experiments [17,18,19] introduced the concept of curvature-induced domain sorting and also highlighted the role of membrane tension in domain stabilization [20]. A significant conceptual advance came with the integration of Ginzburg–Landau-type free energy functionals to describe composition fields, paving the way towards modeling curvature–composition coupling [21]. This was extended to include coupling between composition, curvature, and lipid order parameters [22]. However, a critical limitation persisted: the loss of strict global geometric constraints (constant surface area and volume), leading to non-unique solutions that hampered direct quantitative comparison with experiments on vesicles; thus these solutions could only deal with planar membranes without global area and volume constraints [23]. Recent efforts using phase-field models and dissipative particle dynamics have begun to address these constraints in dynamic simulations [24,25]. This historical context underscores a pivotal gap: the need for a theoretical framework that simultaneously respects the physical constraints of a closed vesicle, accounts for the energetic coupling between membrane curvature and local composition, and can predict both equilibrium shapes and the redistribution of components.

This Special Issue is positioned at the forefront of research addressing this very gap. The collected works represent a multi-faceted attack on the problem of multifunctional membranes, beginning with a theoretical solution to the core modeling challenge and branching out to explore its implications across scales of biological organization.

## 2. A Unifying Theoretical Foundation: Coupling Curvature, Composition, and Constraints

The article by Wu and Ou-Yang [26] delivers a foundational advancement that directly addresses the historical modeling shortfall. They present a generalized Helfrich free-energy framework for multicomponent fluid membranes that is both rigorous and practically applicable. Its significance lies in synthesis: it incorporates a Ginzburg–Landau term for composition heterogeneity, couples it explicitly to membrane curvature, and—crucially—enforces the constraints of fixed total area and volume within the variational calculus. This approach builds upon earlier variational treatments of lipid membranes [27,28] and recent advances in constrained field theory for soft interfaces [29,30,31].

This technical achievement has profound consequences. By deriving new curvature–component coupling Euler–Lagrange equations, the model provides a unified description of equilibrium shapes and the associated spatial sorting of membrane components. These equations generalize earlier results for two-component vesicles [21,32] and provide a continuum counterpart to molecular dynamics studies of lipid domain formation [33,34,35]. The model offers analytical insights, such as the prediction that membrane stability in domains requires distinct bending rigidities among components $k_{A}\neq k_{B}$ and ${\bar{k}}_{A}\neq {\bar{k}}_{B}$, a theoretical anchor for decades of experimental observations on lipid domain coexistence [36,37,38]. Furthermore, the introduction of a new Laplace–Beltrami operator arising from the Gaussian curvature term provides a novel mathematical tool to explain complex phenomena like spontaneous nanotube formation. This mathematical structure connects to earlier geometric treatments of membrane topology [39,40,41] and recent observations of nanotube formation in endoplasmic reticulum and mitochondrial membranes [42,43,44]. This work, building upon earlier investigations into domain shapes and polymer-anchored membranes [45,46,47], achieves more than just extending an equation; it provides a baseline continuum theory against which more detailed microscopic models and experiments can be compared. It aligns with emerging computational frameworks that couple continuum mechanics with discrete molecular details [48,49]. It successfully bridges the gap between the abstract idea of heterogeneity and a quantifiable, predictive physical theory.

## 3. Active Remodeling in a Heterogeneous World: From Mechanisms to Motion

With a robust framework for heterogeneous membranes in place, the next question is how active cellular processes harness and manipulate this complexity. The following two articles delve into dynamic, non-equilibrium mechanisms of membrane reshaping.

Xue and Ma [50] investigate a compelling alternative in yeast endocytosis. Moving beyond the canonical actin-driven model, they demonstrate how boundary lipid flow—powered by myosin motors—can drive membrane tubulation against substantial turgor pressure. By treating the membrane as a viscous–elastic 2D fluid, their model reveals characteristic scaling laws linking pressure to tube geometry. This work integrates principles from active gel theory [51,52] and non-equilibrium membrane hydrodynamics [53,54] and exemplifies a key soft matter principle: the interplay between intrinsic material properties and externally applied stresses. It also resonates with broader research on cellular navigation in complex environments, such as studies on amoeboid [55,56,57,58] and cortical-flow-driven swimming [59], highlighting how physical constraints dictate biological shape change and force generation strategies. Recent studies on cellular mechanosensation and durotaxis further illustrate how membrane–cytoskeleton feedback guides cell migration [60,61,62].

At the molecular scale, Hu and Fu [63] provide a systematic analysis of how protein-induced curvature arises. Their continuum model quantifies how shallowly inserted helical peptides remodel membranes, introducing metrics for quantifying the perturbation area and the extent. This builds on classical work regarding the insertion of an amphipathic helix [64,65,66] and modern molecular simulations of membrane–peptide interactions [67,68,69]. A key finding is the significant role of cooperativity between multiple helices, where spatial arrangement dictates the net mechanical effect. This connects directly to the broader theme of inclusions within membranes. The study of plate-like colloidal islands floating on fluid membranes has shown how elastic interactions can drive organization [70], and theoretical models for membrane-mediated interactions between inclusions have recently been extended to active and fluctuating environments [29,71]. The work of Hu and Fu translates this principle into a biological context, showing how clusters of membrane-active proteins can collectively generate the large-scale curvature needed for processes such as vesicle budding and the direct link to the microdomain and protein–lipid interaction concepts outlined in the historical background.

## 4. Interfacing with the Environment: Adhesion and Energetic Dialogs

The function of a membrane is defined not in isolation but through its interactions. The next articles explore how membrane properties mediate responses to physical and energetic environments.

Mu [72] tackles the classic biophysical problem of cell adhesion, focusing on the red blood cell. The derivation of an adhesive force formula for a biconcave shape, based on the Ouyang–Helfrich equation [41], underscores the importance of specific geometry. This extends earlier adhesion models for vesicles and cells [73,74,75] and complements experimental measurements using micropipette aspiration and optical tweezers [76,77]. The predicted $F\propto D^{-2.5}$ relationship, distinct from a simple sphere, demonstrates how the unique discoid shape, itself a product of membrane–cytoskeleton interactions, fundamentally alters biophysical function. Recent studies on erythrocyte mechanics in microcirculation and malaria infection highlight the physiological relevance of such shape-dependent adhesion [78,79,80]. This work provides a quantitative bridge from membrane mechanics to physiological and biomedical applications like hemostasis.

In a different domain, Lucia and Grisolia [81] address the enigmatic interaction of cells with low-frequency electromagnetic fields (ELF-EMFs). Their thermodynamic model posits the membrane as the primary sensor, where field interactions alter transmembrane energy and matter flows, modulating cellular metabolism. This perspective is supported by experimental evidence for field effects on ion channel gating [82,83], lipid peroxidation [84,85], and membrane protein clustering [86,87]. This view of the cell as an adaptive open system, responding to environmental energy cues, complements the physical picture of adhesion and highlights the membrane’s role as a central processor of diverse external signals. Emerging research on bioelectrical signaling in development and regeneration further underscores the importance of membrane-mediated energy sensing [88,89,90].

## 5. The Ultimate Readout: Linking Membrane Shape to Cellular State

The final article by Guan et al. [91] elevates the discourse from mechanism to meaning. They ask a profound question: can the complex output of the cellular machinery—driven by the very physical processes of heterogeneity, active remodeling, and environmental interaction—be read out in simple geometric terms? Using a vast dataset of *C. elegans* embryonic cells, they demonstrate convincingly that quantitative 3D shape descriptors (e.g., elongation and sphericity) faithfully characterize physiological states like division, migration, and fate specification. This approach is grounded in decades of research on cell morphometrics [92,93,94] and recent advances in machine learning for image-based cell classification [95,96].

This establishes a powerful morpho-functional link, suggesting that the membrane’s shape is not merely a passive outcome but an information-rich signature of the cell’s internal state, echoing earlier ideas in cytology [97,98] and finding support in studies linking nuclear shape to gene expression [99,100] and cell shape to stem cell differentiation [101,102]. This has transformative implications, potentially allowing physical shape to serve as a biomarker for health and disease. It also creates a conceptual bridge to tissue-scale physiology; the organization of tissues, including pathological architectures like tumor stroma [103], emerges from the collective behavior and adhesion of cells—properties rooted in their membrane mechanics. Computational models of tissue morphogenesis increasingly incorporate cell membrane mechanics as a key driver [104,105,106]; thus, the principles governing individual membrane shape may scale to influence collective tissue behavior.

## 6. Synthesis and Outlook: From Descriptive to Predictive Membrane Science

The trajectory revealed by this Special Issue marks a transition in membrane biophysics from descriptive models of idealized systems to predictive frameworks for complex, living matter. The theoretical work by Wu and Ou-Yang [26] solves a key piece of this puzzle, providing a mathematically sound foundation for heterogeneous membranes. The subsequent articles explore the consequences: how active processes leverage this heterogeneity, how membranes interface with the world, and how their final shape encodes functional information.

Looking forward, this foundation enables several exciting frontiers to be investigated:

- Integrative Multi-scale Simulations: Coupling the new continuum framework [26] with coarse-grained molecular dynamics [46,47] will enable unprecedented simulations of protein aggregation [63] in domains and large-scale membrane remodeling in complex environments [50,107,108]. Recent efforts in hybrid simulation methodologies show great promise in this direction [49,109,110].
- Engineering with Heterogeneity: Principles of curvature–composition coupling [26] and inclusion interactions [70,111,112,113,114] with chemical gradients [115] can guide the design of “smart” drug delivery vesicles [116] that target specific tissues or release cargo in response to membrane phase changes. Advances in synthetic biology and biomimetic materials are already exploiting these principles [117,118,119].
- Machine Learning-Enhanced Biophysics: The shape descriptors from Guan et al. [91] are perfect for integration with AI to diagnose cellular pathology from standard microscopy images, creating a direct pipeline from a physical principle to a clinical tool. The field of computational pathology is rapidly evolving in this direction [120,121,122].
- Targeting the Physical Microenvironment: Understanding how membrane-level mechanics influence cell adhesion [72] and signaling can inform novel therapies that modulate the tissue architecture in diseases like cancer [103,123] and fibrosis [103]. The emerging field of mechanomedicine is built upon this premise [124,125,126].

## 7. Conclusions

The study of biological membranes has progressed from visualizing simple vesicles to grappling with the sophisticated, heterogeneous, and active composite material that defines a cell. This Special Issue exemplifies the power of the soft matter approach and captures a pivotal moment in this journey to unravel the design principles of biological membranes. By synthesizing theoretical innovation, mechanistic discovery, and quantitative biophysical analysis, the collected articles advance our understanding of how molecular and physical interactions give rise to membrane function, advances which are built upon a rich foundation of prior work in membrane mechanics and cell biophysics. The insights contained herein are not merely academic; they provide the fundamental design principles needed to engineer next-generation biomaterials, therapeutic strategies, and diagnostic platforms. As we continue to unravel the complexities of the membrane, we move closer to harnessing its ingenuity to advance human health.

As the field of soft matter continues to expand and intersect with biology, medicine, and engineering, the study of biological membranes will remain a central and fertile ground for discovery. The insights gained not only satisfy fundamental scientific curiosity but also provide the conceptual tools needed to engineer novel solutions in healthcare, from smart drug delivery systems to advanced diagnostic tools. We hope this collection will inspire continued interdisciplinary dialog and innovation, driving forward the exciting convergence of soft matter physics and the life sciences.
